# Development and Validation of a GC-MS Method for the Detection and Quantification of Clotiapine in Blood and Urine Specimens and Application to a Postmortem Case

**DOI:** 10.1155/2015/972480

**Published:** 2015-07-07

**Authors:** Giulio Mannocchi, Flaminia Pantano, Roberta Tittarelli, Miriam Catanese, Federica Umani Ronchi, Francesco Paolo Busardò

**Affiliations:** Department of Anatomical, Histological, Forensic and Orthopaedic Sciences, Sapienza University of Rome, Viale Regina Elena 336, 00161 Rome, Italy

## Abstract

*Introduction*. Clotiapine is an atypical antipsychotic of the dibenzothiazepine class introduced in a few European countries since 1970, efficient in treatment-resistant schizophrenic patients. There is little published data on the therapeutic and toxic concentrations of this drug. *Aims*. The aim of the present study is the development and validation of a method that allows the detection and quantification of clotiapine in blood and urine specimens by gas chromatography-mass spectrometry (GC-MS). *Methods*. Validation was performed working on spiked postmortem blood and urine samples. Samples were extracted with liquid-liquid extraction (LLE) technique at pH 8.5 with n-hexane/dichloromethane (85/15 v/v) and analysis was followed by GC-MS. Methadone-d9 was used as internal standard. *Results*. The limit of detection (LOD) was 1.2 and 1.3 ng/mL for urine and blood, respectively, while the lower limit of quantification (LLOQ) was 3.9 and 4.3 ng/mL, respectively. Linearity, precision, selectivity, accuracy, and recovery were also determined. The method was applied to a postmortem case. The blood and urine clotiapine concentrations were 1.32 and 0.49 *μ*g/mL, respectively. *Conclusions*. A reliable GC-MS method for the detection and quantification of clotiapine in blood and urine samples has been developed and fully validated and then applied to a postmortem case.

## 1. Introduction

**Figure 1:**
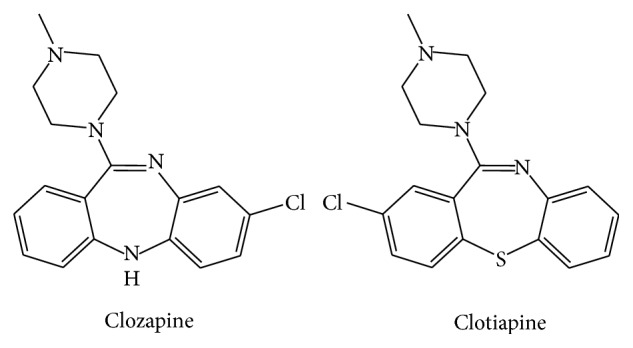
Chemical structures of clozapine and clotiapine.

Clotiapine has been described as both typical and atypical antipsychotic in literature. It belongs to the dibenzothiazepine chemical class and was introduced in a few European countries since 1970. It is characterized by high incidence of extrapyramidal side effects, but some authors [1] underlie its efficacy in treating resistant schizophrenic patients. Its chemical structure is similar to that of clozapine; structural similarities are shown in Figure 1.

The mechanism of action of this neuroleptic is due to downregulation of 5HT2-receptors [2]. Clotiapine shows also great affinity for 5HT-3,6 receptors. It seems also that it acts on dopamine receptors [3] and as an antagonist to histamine, norepinephrine, serotonin, and dopamine in animal models [4]. It has a rapid onset of action and it is characterized by marked sedative effects; therefore it is used to manage acute and chronic schizophrenia, agitation, bipolar, panic, and sleep disorders [5]. Moreover, it is also used for the treatment of drug withdrawal symptoms [6], as suggested by animal studies regarding heroin addiction. The effects noticed following clotiapine administration were antidopaminergic, antimuscarinic, and antiadrenergic that could counteract withdrawal symptoms [7]. This drug is able to produce anticholinergic adverse reactions in the 25% of cases as reported by Cicero et al. [8]. In literature the possibility of an increased risk of ventricular arrhythmias, sudden cardiac death, and drug induced long QT interval is reported [9, 10]. Moreover, clotiapine has been linked with fatalities in association with other substances such as clomipramine, [11] alcohol, [12] citalopram, and mirtazapine [13]. Overdose in a female infant, accompanied by central nervous system (CNS) depression and managed by supportive treatment without sequelae, has also been described [14].

Toxicological investigation in the determination of the cause of death plays a crucial role. In cases of deaths that are not directly attributed to intoxication itself but related to pharmacological mechanisms that could contribute to the development of complex multifactorial syndromes, the identification of involved drugs is important to assist in the determination of the cause of death. In this paper, a method for detection and quantification of clotiapine in blood and urine samples was developed and fully validated according to the guidelines of Peters et al. [15]. The method was then applied to a postmortem case.

## 2. Case Report

A 45-year-old man (185 cm of body height and 95 kg of body weight) died in bed in his private flat. Relatives could provide only limited information about the circumstances of death, but positive anamnesis about mental illness (schizophrenia) was reported. A complete autopsy and a full toxicological analysis were performed.

### 2.1. Autopsy Results

**Figure 2:**
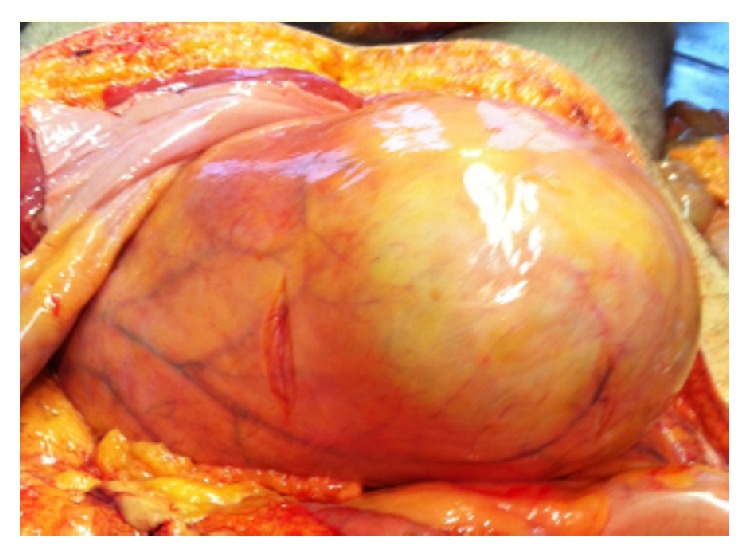
Internal examination: overdistended bladder containing 3.7 L of urine.

The external examination revealed only a remarkable abdominal swelling. The internal examination showed a moderate pulmonary oedema and the presence of massive urinary retention: the bladder contained 3.7 L of urine (Figure 2). The kidneys (weight 270 gr, the right, and 260 gr, the left) were markedly congested.

The pathological evidences in combination with the referred schizophrenia suggested that a pharmacological mechanism could have contributed to the massive urinary retention.

## 3. Experimental

### 3.1. Chemicals, Reagents, and Samples

Peripheral blood (15 mL), urine (40 mL), and gastric contents (40 mL) were collected during autopsy and stored at −20°C. The internal standard selected for quantitative determination of clotiapine in biological samples was methadone-d9, purchased from Cerilliant (Round Rock, TX). Reference standards for clotiapine were purchased from ALSACHIM (Illkirch-Graffenstaden, France). Ethyl acetate, n-hexane, and dichloromethane were acquired from Carlo Erba (Milan, Italy). Ultrapure distilled and deionized water was homemade (Millipore-Helix 70) and filtered prior to use.

### 3.2. Preparation of Standards

Blank urine and blood samples, collected from autopsies performed in our department, were used to prepare the standard calibration curves, obtained by fortification with certified standard of clotiapine in range of concentration from 10 to 2000 ng/mL. 500 ng of methadone-d9 was added, in each sample, as internal standard. The samples used to determine the accuracy, precision, and recovery of the method were prepared as described above.

### 3.3. Extraction Procedure

Fortified blank urine and blood were extracted according to the following procedure: to 1 mL of each sample, 1 mL of deionized water and 500 ng of methadone-d9 were added. Samples were extracted at pH 8.5 (adding 50 mg of solid HCO_3_^−^/CO_3_^−−^ buffer) with 4 mL of extraction solution (n-hexane/dichloromethane (85/15 v/v)) [16] after 15 minutes of steering. After centrifugation (4000 rpm, 3 min) the organic layer was evaporated to dryness under a gentle stream of nitrogen. The residue was reconstituted in 50 *μ*L of ethyl acetate. Gastric content sample was extracted according to the above mentioned procedure.

### 3.4. Instrumentation and Conditions

Analysis was carried out on a gas chromatography instrument Agilent HP 7028A GC coupled with an Agilent MSD 5975. The capillary column used was an HP-5MS (17 m × 0.25 mm I.D. coated with a 0.25 *μ*m film). The GC conditions were as follows: the column temperature was programmed from 125°C to 290°C with an increase of 10°C/min; the injection port and the transfer line temperature were 270°C; helium was used as carrier gas at flow rate of 1 mL/min; split ratio was 15 : 1. The mass analyzer operated by electron impact (70 eV) in selected ion monitoring (SIM). Quantitative analysis was carried out recording ions *m*/*z* 209-244-343 for clotiapine and *m*/*z* 78-165-303 for methadone-d9. The underlined ions were used for quantitative analysis.

### 3.5. Method Validation

The method was validated on urine and blood specimens, according to the procedure of Peters et al. [15] “for single case analysis or for analysis of rare analytes”; in addition, an extra parameter, recovery, was determined as well.

*Selectivity* was evaluated by analysis of six sources of blank matrices (blood and urine samples collected from autopsies) processed with 1 zero sample (blank matrix with internal standard), verifying the absence of signal interferences. The* Calibration Model* was selected by analyzing 6 concentration levels, from 10 ng/mL to 2000 ng/mL, and each level was evaluated in triplicate according to a linear model.* Accuracy* (bias) and* precision* were estimated from the analysis of quality control (QC) samples at low (close to lower limit of quantification (LLOQ)) and high concentration (2000 ng/mL), in five replicates for each level. The acceptance criterion for bias was within ±15% of nominal value (±20% close to LLOQ), for precision was within ±15% relative standard deviation (RSD) (20% close to LLOQ). The LOD (*Limit of Detection*) was determined by analysis of spiked samples with decreasing level of concentration of the analyte. For LOD a value of signal-to-noise ratio equal to or greater than three (*S*/*N* ≥ 3) was chosen. The LLOQ was determined by analysis of fortified samples with decreasing level of concentration of the analyte. For LLOQ a value of signal-to-noise ratio equal to or greater than ten (*S*/*N* ≥ 10) was chosen.* Recovery* was calculated by analyzing extracted spiked samples at high and low concentration in relationship with the curve calibration, compared with the control samples.

### 3.6. Determination of Creatinine Concentrations and Creatinine-Normalization

The clotiapine concentrations in urine samples were creatinine-normalized [17]. The creatinine (CR) concentration was determined by using an ILab 650 analyzer. Clotiapine concentrations in urine were CR-normalized based on a previously published method [17] using the following equation: concentration CR-normalized = clotiapine concentration · (CR reference)/(CR sample). The CR reference was 100 mg/dL [17].

## 4. Results

### 4.1. Validation Results

The method was validated by investigating the following parameters: linearity, selectivity, identification of LOD and LLOQ, precision, accuracy, and recovery. The results are shown in Table 1.

The calibration plots showed good linearity, with a determination coefficient of 0.9996 and 0.9997 in blood and urine, respectively.

### 4.2. Toxicological Findings

Toxicological screening, performed with immunochemical technique, revealed the absence of the most common abuse substances. Alcohol intake was excluded by performing HS-GC-FID analysis. A GC-MS screening analysis to evaluate the presence of drugs that could contribute to urinary retention (according to the anamnestic data) was carried out in blood and urine. Clotiapine was found in both matrices.

**Figure 3:**
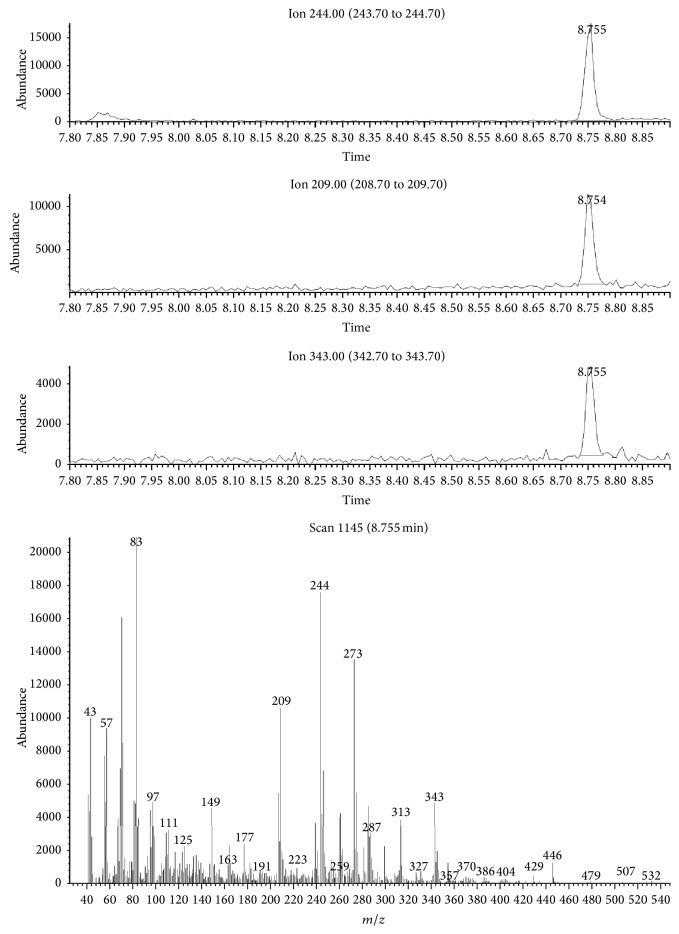
Full scan mode chromatogram and mass spectra obtained from blood sample analysis.

**Figure 4:**
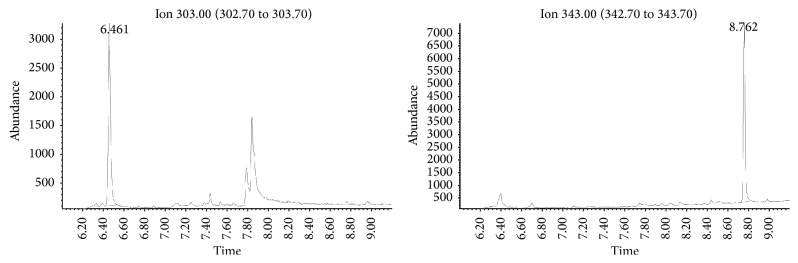
SIM mode chromatogram showing methadone-d9 (*m*/*z*: 303) and clotiapine (*m*/*z*: 343) in blood sample.

Afterwards, toxicological analysis, using the validated method described here, retrieved the following concentrations: 1.32, 0.49, and 1.85 *μ*g/mL in peripheral blood, urine, and gastric contents, respectively. No other drugs or alcohol was detected in biological samples. Full scan mode chromatograms and mass spectra as well as SIM mode chromatograms, indicating clotiapine and methadone-d9, obtained from the analysis of the blood sample, are given in Figures 3 and 4, respectively. In Figure 3, the peaks of extracted ions that were used for qualitative and quantitative analysis of clotiapine (*m*/*z* 209-244-343) are presented. The retention time of clotiapine as shown in the same figure is 8.755 minutes. In Figure 4, SIM mode chromatograms of analytes of interest (*m*/*z*: 303 for methadone-d9 and *m*/*z*: 343 for clotiapine) are given. The retention times recorded for methadone-d9 and clotiapine are 6.461 and 8.762, respectively.

## 5. Discussion and Conclusions

A GC-MS method for the detection and quantification of clotiapine in blood and urine samples has been developed, validated, and then successfully applied to real samples. The application of the method to a fatal case involving a 45-year-old schizophrenic subject allows us to identify clotiapine at very high concentrations in blood, urine, and gastric content. The presence of clotiapine in gastric contents suggests an oral intake. Because of the remarkable sedative characteristics of this substance, the deceased could not probably be in a state to be aware and face the symptoms. Particular caution is recommended when treating patients with urinary retention (especially due to prostatic hypertrophy). Moreover, it is not advisable to coadminister anticholinergic agents and clotiapine, since the latest could lead to harmful effects including vision disorders, constipation, urinary retention, possible increase in intraocular pressure, and mouth dryness.

Lethal dose 50 (LD 50) after oral intake in the mouse, rat, and Guinea pig is 272, 480, and 154 mg/kg, respectively. However, therapeutic, toxic, and fatal doses in human are still non-well-established [18–21]. The plasma clotiapine concentrations found in twelve clotiapine-treated patients ranged from 0.006 to 0.155 mg/L (mean 0.046 mg/L) while the concentrations reported in seven autopsy cases ranged from 0.022 to 0.341 mg/L (mean 0.123 mg/L) [16]. However, in the latest study clotiapine was only a contributing factor in the cause of death of multidrug intoxications; none of the deaths was attributed to clotiapine poisoning.

In this case the cause of death taking into consideration the autopsy and toxicological findings was attributed to postrenal acute kidney injury due to a severe urinary retention (3.7 L of urine in the bladder).

## Figures and Tables

**Table 1:** Method validation parameters and data.

	Selectivity	Linearity (*R* ^2^)	Accuracy (bias%)		Precision (RSD%)		LOD	LLOQ	Recovery (%)	
			Low	High	Low	High			Low	High
Urine	No interference signals	0.9997	18	−0.6	14	3.2	1.2 ng/mL	3.9 ng/mL	103	99

Blood	No interference signals	0.9996	−18	−0.5	19	3.0	1.3 ng/mL	4.3 ng/mL	93	95
